# Ni_0.5_Cu_0.5_Co_2_O_4_ Nanocomposites, Morphology, Controlled Synthesis, and Catalytic Performance in the Hydrolysis of Ammonia Borane for Hydrogen Production

**DOI:** 10.3390/nano9091334

**Published:** 2019-09-18

**Authors:** Yufa Feng, Jin Zhang, Huilong Ye, Liling Li, Huize Wang, Xian Li, Xibin Zhang, Hao Li

**Affiliations:** 1 School of Chemistry and Materials Engineering, Huizhou University, Huizhou 516007, China; yufafeng@126.com (Y.F.); eeedwardjin@163.com (J.Z.); yehuilong6364@163.com (H.Y.); whz@hzu.edu.cn (H.W.); lixian2020@126.com (X.L.); zxbin1@163.com (X.Z.); 2 Department of Pharmacy, Huizhou Health Sciences Polytechnic, Huizhou 516025, China

**Keywords:** nanocomposites, heterogeneous catalysis, hydrogen production, ammonia borane, nanostructures

## Abstract

The catalytic hydrolysis of ammonia borane (AB) is a promising route to produce hydrogen for mobile hydrogen‒oxygen fuel cells. In this study, we have successfully synthesized a variety of Ni_0.5_Cu_0.5_Co_2_O_4_ nanocomposites with different morphology, including nanoplatelets, nanoparticles, and urchin-like microspheres. The catalytic performance of those Ni_0.5_Cu_0.5_Co_2_O_4_ composites in AB hydrolysis is investigated. The Ni_0.5_Cu_0.5_Co_2_O_4_ nanoplatelets show the best catalytic performance despite having the smallest specific surface area, with a turnover frequency (TOF) of 80.2 mol_hydrogen_·min^−1^·mol^−1^_cat_. The results reveal that, in contrast to the Ni_0.5_Cu_0.5_Co_2_O_4_ nanoparticles and microspheres, the Ni_0.5_Cu_0.5_Co_2_O_4_ nanoplatelets are more readily reduced, leading to the fast formation of active species for AB hydrolysis. These findings provide some insight into the design of high-performance oxide-based catalysts for AB hydrolysis. Considering their low cost and high catalytic activity, Ni_0.5_Cu_0.5_Co_2_O_4_ nanoplatelets are a strong candidate catalyst for the production of hydrogen through AB hydrolysis in practical applications.

## 1. Introduction

With the ever-increasing consumption of fossil fuels, many global environmental issues have emerged, such as global warming, acid rain, severe smog, etc. [1]. Accordingly, it is an urgent task to find new green and renewably energy sources to replace fossil fuels. Hydrogen is considered as a suitable fossil-fuel replacement candidate due to its ability to generate a huge amount of heat with zero CO_2_ emissions [2]. However, its safe and effective storage and transportation are still technical challenges that need to be addressed for its successful large-scale application [3]. As a hydrogen storage material, chemical hydrides have received considerable attention in recent years. Among them, ammonia borane (NH_3_BH_3_, AB) has a high hydrogen content (19.6 wt %) [4,5], high stability in both solid state and aqueous solution at room temperature, and no toxicity [6]. In addition, AB can be dehydrogenized via hydrolysis [7], solvolysis [8], and pyrolysis [9]. Among these approaches, hydrolysis is the most promising way to produce hydrogen through the following reaction:

H_3_NBH_3_ (aq) + 2H_2_O (l) →NH_4_^+^+ (aq) + BO_2_^−^ (aq) + 3H_2_ (g). (1)

Although the reaction is thermodynamically feasible, the AB hydrolysis reaction is very slow [10]. Thus, it is important to find an efficient catalyst to accelerate the hydrolytic process. Generally, the heterogeneous catalysts for AB hydrolysis include noble-metal-based catalysts, such as Pt [11], Pd [12], Ru [13], PtNi [6], PtCu [14], PdNi [15], and low-cost metal-based catalysts, including Ni [16], Co [17], Cu [18], CoNi [19] and CoCu alloys [20]. Although the former manifest superior catalytic activity, their industrial-scale application is restricted by the high cost. On the other hand, the catalytic performance of non-noble-metal-based catalysts is not high enough to enable fast hydrogen production in practical applications despite their low cost. Therefore, it is crucial to develop other types of inexpensive catalysts with superior catalytic activity. Over the past several years, non-noble-metal oxide-based nanocomposites have emerged as a new type of catalyst for AB hydrolysis [21,22,23]. For example, Yamada et al. successfully controlled a series Cu_2_O/Co_3_O_4_ composites by adjusting the reaction conditions. Under optimal conditions, the nanocomposite of Cu_2_O and Co_3_O_4_ demonstrates high catalytic behavior, with a hydrogen evolution rate of 0.78 mmol_hydrogen_·s^−1^·g_cat_^−1^ [21]. CuO‒NiO nanocomposites have been proven by Yen et al. to be a robust catalyst towards AB hydrolysis with a TOF of 60 mol_hydrogen_·mol_Cu_^−1^·min^−1^ [22]. Feng et al. prepared a particulate nanocomposite of CuO and CoO supported on grapheme oxide, which exhibit high catalytic activity with a TOF of 70.0 mol_hydrogen_·mol_cat_^−1^·min^−1^ [23]. These successful examples have demonstrated that the catalytic activity in AB hydrolysis can be remarkably improved by joining different oxide components together into composites. However, it is still unclear what role each component plays and why there is a synergistic effect between the different components. To clarify these issues, further investigation is still necessary. 

On the other hand, the catalytic performance of nanocatalysts was found to be strongly dependent on their morphology [24]. Yamada et al. synthesized Co_3_O_4_ nanoparticles, nanoplatelets, and nanocubes and investigated their catalytic behavior in AB hydrolysis [25]. They found that Co_3_O_4_ nanoplatelets exhibited the best catalytic performance among those catalysts. Yao et al. prepared mesoporous CuO with diverse morphologies via a facile wet-chemical method and then used the mesoporous CuO as robust catalysts in hydrogen production by methanolysis of AB [26]. The flower-like mesoporous CuO nanocatalyst showed the highest catalytic activity, with a TOF of 2.41 mol_hydrogen_·min^−1^·mol^−1^_cat_, compared with the nanoplatelet-like, bundle-like, and dandelion-like CuO. Very recently, Zheng et al. prepared CuCoO nanocubes and nanoparticles supported on reduced graphene oxide [27]. They found that the CuCoO nanocubes exhibited much higher catalytic activity in AB hydrolysis than CuCoO nanoparticles. These findings clearly indicate that the activity of the oxide-based nanocatalysts in AB hydrolysis is influenced by their morphology. However, the reasons for these observations are still not well understood. 

Motivated by these findings, in this work, we developed a facile route to prepare differently shaped Ni_0.5_Cu_0.5_Co_2_O_4_ nanocomposites, namely nanoparticles, nanoplatelets, and urchin-like microspheres composed of nanowires. So far, such Ni_0.5_Cu_0.5_Co_2_O_4_ nanocomposites have not been reported in the literature. Additionally, their catalytic activity in AB hydrolysis at room temperature was investigated. 

## 2. Experimental

### 2.1. Synthesis of Catalysts

All chemical reagents were obtained from commercial suppliers and used without further purification. To prepare Ni_0.5_Cu_0.5_Co_2_O_4_ nanoparticles, 2.0 mmol CoSO_4_·7H_2_O (Tianjin Baishi Chemical Reagent Co.Ltd., Tianjin, China), 0.5 mmol CuSO_4_·5H_2_O (Tianjin Baishi Chemical Reagent Co.Ltd., Tianjin, China), and 0.5 mmol NiSO_4_·6H_2_O (Tianjin Baishi Chemical Reagent Co.Ltd., Tianjin, China) were dissolved in 40 mL of deionized water with magnetic stirring. Then, 20 mL of sodium citrate (Tianjin Baishi Chemical Reagent Co.Ltd., Tianjin, China) solution (0.1 M) were added, drop by drop, to the above solution to obtain a purple solution. Subsequently, 20 mL of NaOH (Taishan Yueqiao Chemical Reagent plastics Co.Ltd., Jiangmen, China) solution (5 M) was slowly added into the mixed solution, which changed the color from purple to mazarine. After stirring for 30 min, the aqueous solution was subjected to hydrothermal treatment at 120 °C for 8 h in a Teflon-lined autoclave. Then, the collected powder was cleaned with water and annealed at 500 °C for 2 h. The synthesis of Ni_0.5_Cu_0.5_Co_2_O_4_ nanoplatelets is similar to that of the Ni_0.5_Cu_0.5_Co_2_O_4_ nanoparticles mentioned above, except that the sodium citrate was replaced by ethylenediamine tetraacetic acid disodium salt (EDTA-2Na) (Taishan Yueqiao Chemical Reagent plastics Co.Ltd., Jiangmen, China). To prepare the urchin-like Ni_0.5_Cu_0.5_Co_2_O_4_ microspheres, polyethylene glycol 2000 (1 g) (Tianjin Damao Chemical Reagent Co.Ltd., Tianjin, China) was used instead of sodium citrate, and urea (6 g) (Tianjin Yongda Chemical Reagent Co.Ltd., Tianjin, China) was used instead of NaOH.

### 2.2. Characterizations

The X-ray powder diffraction (XRD) patterns were obtained using a Rigaku D/Max-1200X diffractometer (Rigaku Corp., Tokyo, Japan) with Cu Kα radiation (λ = 1.5406 Å and 40 kV, 200 mA). The morphology of the samples was examined by a field emission scanning electron microscope (FE-SEM) on a Hitachi Su-8010FE-SEM microscope (Hitachi Ltd., Tokyo, Japan). Transmission electron microscopy (TEM) and high-resolution TEM (HRTEM) images were obtained on a FEI Tecnai G2 F20 S-TWIN transmission electron microscope (FEI Co., Hillsboro, OR, USA). Fourier transform infrared (FT-IR) spectroscopy was used to record the FT-IR spectrum from 4000 to 400 cm^−1^ on a Bruker TENSOR 27 FT-IR spectrometer (Bruker Optics GmbH, Ettlingen, Germany). The nitrogen adsorption‒desorption isotherm and the Brunauer–Emmett–Teller (BET) surface areas of the products were measured using a Quantachrome 3H-2000 nitrogen adsorption analyzer (Quantachrome Instruments, Boynton Beach, FL, USA). X-ray photoelectron spectroscopy (XPS) was performed on a Kratos Axis Ultra DLD X-ray photoelectron spectrometer (Kratos Analytical Ltd., Manchester, UK) with Al Kα radiation. The temperature-programmed reduction (TPR) was performed on a Micromeritics AutoChem II 2920 chemisorption analyzer (Micromeritics Instrument Corp., Norcross, GA, USA) at a linear heating rate (10 °C/min) with a flow of 10% H_2_ in argon at a flow rate of 40 mL/min.

### 2.3. Catalytic Tests

Unless specified, the catalytic performance was tested at 298 K. In a typical process, 5.0 mg of catalyst powder was dispersed in 5.0 mL of deionized water with ultrasonication. Subsequently, 15 mL of a mixture solution containing 3 mmol (0.0926 g) of AB and 0.8 g of NaOH was poured into the vessel. The volume of the generated gas was monitored by recording the displacement of water in the gas burette. 

## 3. Results and Discussion

### 3.1. Characterization of the Catalysts

**Figure 1:**
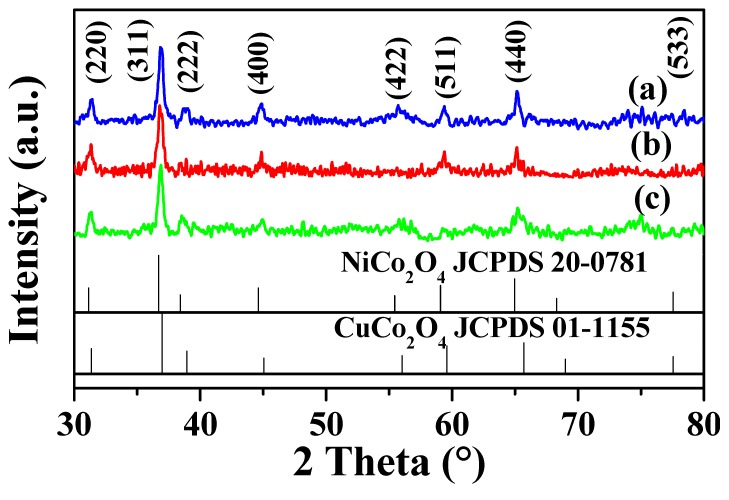
XRD patterns of the Ni_0.5_Cu_0.5_Co_2_O_4_ nanoparticles (**a**), urchin-like microspheres (**b**), and nanoplatelets (**c**).

The XRD patterns of the differently shaped Ni_0.5_Cu_0.5_Co_2_O_4_ nanocomposites are displayed in Figure 1. For comparison, the standard patterns of spinel CuCo_2_O_4_ (JCPDS01-1155) and NiCo_2_O_4_ (JCPDS20-0781) are also shown in Figure 1. Note that the peak position or peak intensity of the standard patterns of spinel CuCo_2_O_4_ and NiCo_2_O_4_ are quite similar, which is to be expected since spinel CuCo_2_O_4_ and NiCo_2_O_4_ have almost the same crystal structure. In addition, the radius of the Cu atom is close to that of the Ni atom. Thus, the replacement of Cu with Ni, and vice versa, will not result in a pronounced structural change. All the diffraction peaks of the urchin-like microspheres, nanoplatelets, and nanoparticles match well with those of the standard patterns, which were ascribed to the (220), (311), (222), (400), (422), (511), (440), and (533) planes of the spinel crystal structure of CuCo_2_O_4_ and NiCo_2_O_4_. It should, however, be pointed out that it is difficult to distinguish from the XRD results whether our Ni_0.5_Cu_0.5_Co_2_O_4_ samples are CuCo_2_O_4_/NiCo_2_O_4_ nanocomposites or just a physical mixture of CuCo_2_O_4_ and NiCo_2_O_4_, considering that the CuCo_2_O_4_ and NiCo_2_O_4_ can hardly be distinguished by XRD analysis alone.

**Figure 2:**
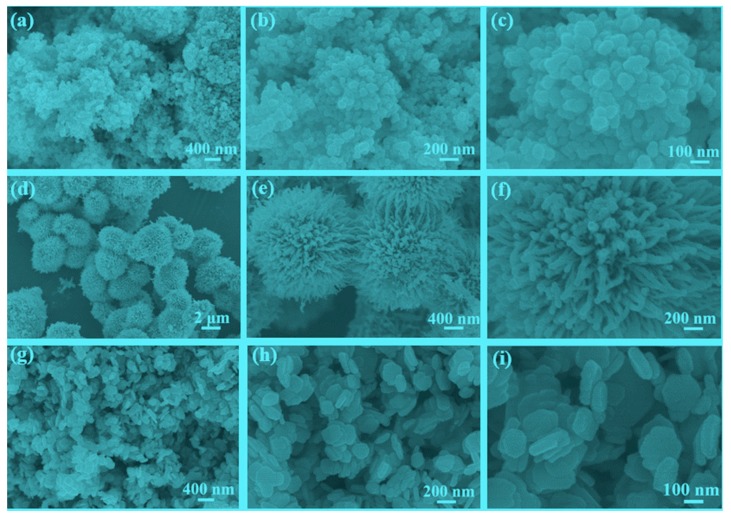
SEM images of the Ni_0.5_Cu_0.5_Co_2_O_4_ nanoparticles (**a**–**c**), Ni_0.5_Cu_0.5_Co_2_O_4_ microspheres (**d**–**f**), and Ni_0.5_Cu_0.5_Co_2_O_4_ nanoplatelets (**g**–**i**).

SEM images of Ni_0.5_Cu_0.5_Co_2_O_4_ nanocomposites are displayed in Figure 2. The images of Ni_0.5_Cu_0.5_Co_2_O_4_ nanoparticles in Figure 2a–c indicate that they were successfully synthesized. The nanoparticles of different size are homogeneously distributed, with a typical size of 50 nm. The urchin-like microspheres, which were fabricated by adjusting the synthetic conditions, are shown in Figure 2d–f, and have a diameter of approximately 3 μm. These microspheres are composed of numerous nanowires with irradiation arrangement. These nanowires have a uniform diameter of approximately 40 nm. The images in Figure 2g–i indicate that abundant regularly shaped Ni_0.5_Cu_0.5_Co_2_O_4_ nanoplatelets were successfully obtained. The typical size and thickness of these nanoplatelets are 200 and 35 nm, respectively. For comparison, the SEM images of the mixture of CuCo_2_O_4_ and NiCo_2_O_4_, and EDS patterns of some selected nanoplatelets are shown in Figure S1. It is found that the morphology of the mixture of CuCo_2_O_4_ and NiCo_2_O_4_ is similar to that Ni_0.5_Cu_0.5_Co_2_O_4_ nanoplatelets. However, the EDS results clearly suggest that separated CuCo_2_O_4_ and NiCo_2_O_4_ nanoplatelets coexist in the mixture sample. 

**Figure 3:**
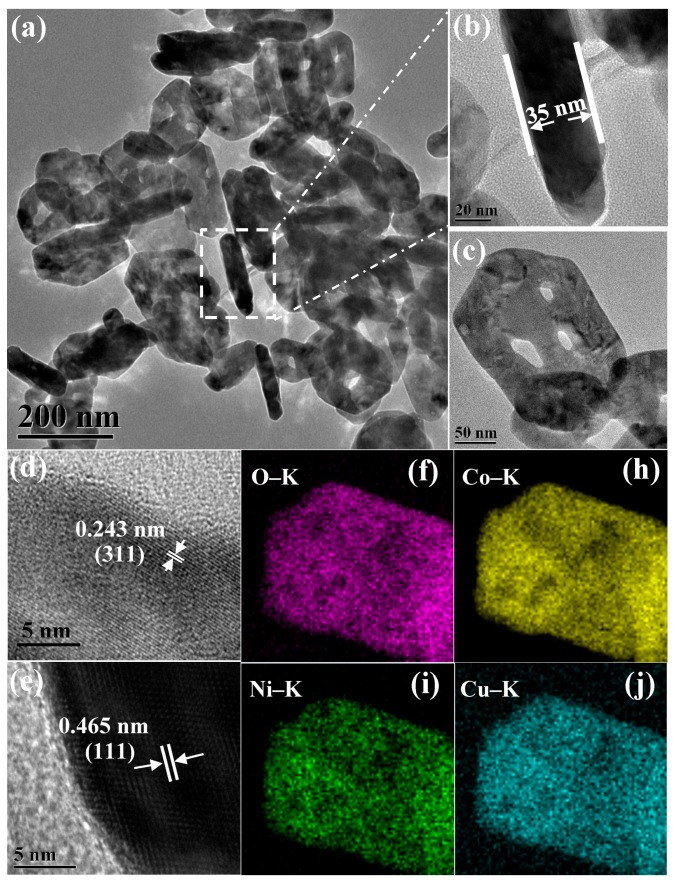
TEM images of Ni_0.5_Cu_0.5_Co_2_O_4_ nanoplatelets (**a**–**c**), HRTEM images of Ni_0.5_Cu_0.5_Co_2_O_4_ nanoplatelets (**d**,**e**), and the elemental mapping of a piece of Ni_0.5_Cu_0.5_Co_2_O_4_ nanoplatelet (**f**,**h**–**j**).

The TEM and HRTEM images of the as-prepared Ni_0.5_Cu_0.5_Co_2_O_4_ nanoplatelets, displayed in Figure 3a–e, further confirm the architecture of the nanoplatelets with a thickness of about 35 nm, which is in line with the SEM observation. The lattice fringes of 0.243 nm and 0.465 nm correspond to the (311) and the (111) interplanar spacings of Ni_0.5_Cu_0.5_Co_2_O_4_, respectively. The TEM/HRTEM images of the urchin-like Ni_0.5_Cu_0.5_Co_2_O_4_ microspheres and the Ni_0.5_Cu_0.5_Co_2_O_4_ nanoparticles, given in Figure S2, reveal that the samples are polycrystalline and the space of the lattice fringes agree with the interplanar spacings of Ni_0.5_Cu_0.5_Co_2_O_4_. Since CuCo_2_O_4_ and NiCo_2_O_4_ have a similar crystal structure, it is hard to distinguish, by TEM, HRTEM, and XRD analysis whether our samples are CuCo_2_O_4_/NiCo_2_O_4_ nanocomposites or just a mixture of CuCo_2_O_4_ and NiCo_2_O_4_. To make this distinction, we performed a two-dimensional elemental analysis on a piece of nanoplatelet; the elemental mapping results are shown in Figure 3f,h–j. It was found that the elements of Co, Ni, Cu, and O are uniformly distributed in the nanoplatelet, thus confirming that our samples are CuCo_2_O_4_/NiCo_2_O_4_ nanocomposites rather than a physical mixture of the two compounds. As we can see in the experimental section, the designed molar ratio of CuCo_2_O_4_ to NiCo_2_O_4_ is 1:1. The atomic ratio of Cu, Ni, and Co in the composites by ICP-MASS is 1:0.91:3.85 for nanoplatelets, 1:0.90:3.86 for microspheres, and 1:0.95:3.92 for nanoparticles, respectively. All the ratios are close to the expected value of 1:1:4.

**Figure 4:**
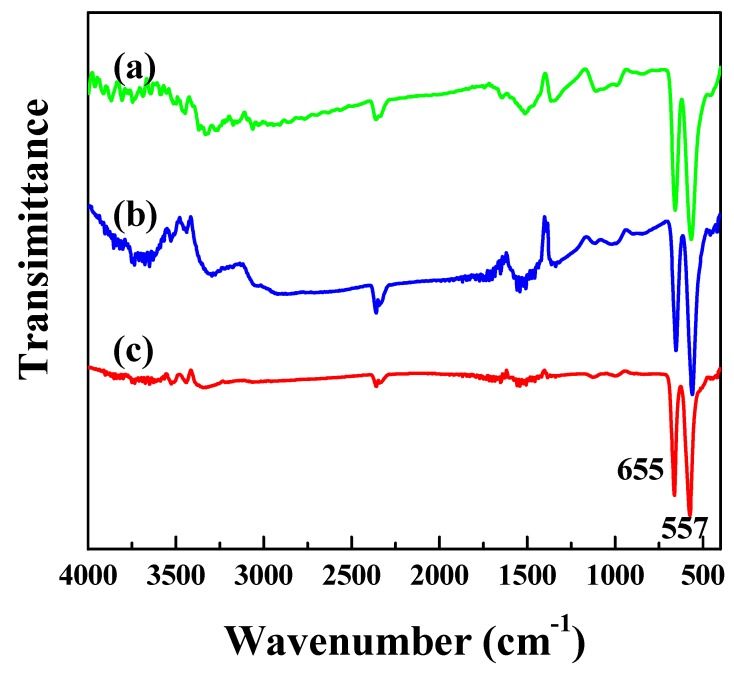
FT-IR spectra of Ni_0.5_Cu_0.5_Co_2_O_4_ nanoparticles (**a**), urchin-like microspheres (**b**), and nanoplatelets (**c**).

FT-IR spectroscopy analysis was performed on Ni_0.5_Cu_0.5_Co_2_O_4_ nanocomposites to determine the functional groups in the three samples, as shown in Figure 4. The peaks at around 3438 and 1634 cm^−1^ were assigned to both ν_s_(O-H), and ν_as_(O-H) vibrations and δ(H-O-H) vibrations of hydrated water, respectively. The peak at 2358 cm^−1^ was ascribed to the absorption of CO_2_. According to the literature [28,29,30], CuCo_2_O_4_ and NiCo_2_O_4_ have the same spinel structure, showing in their fingerprint region two intense peaks between 400 and 700 cm^−1^ at approximately 650 and 550 cm^−1^, attributed to M^2+^- O^2−^ (M = Cu or Ni) and Co^3+^- O^2−^, respectively. In this study, two strong peaks at 655 and 557 cm^−1^ were observed in the FT-IR spectra of the three samples, confirming the formation of the spinel structure of Ni_0.5_Cu_0.5_Co_2_O_4_ nanocomposites.

The surface area of a heterogenous catalyst has a significant impact on its catalytic performance [31]. In general, a larger specific surface area will lead to higher catalytic activity. However, there is no direct relationship between the activity and the surface area in many cases [32]. The N_2_ adsorption‒desorption isotherms of the three samples are shown in Figure S3. Based on the classification of the International Union of Pure and Applied Chemistry, the presence of a hysteresis loop in Figure S3 indicated that the isotherm curves correspond to the typical Langmuir type IV isotherm, which is indicative of the existence of mesoporous pores in the samples. No platform appeared around the high relative pressure (P/P_0_) according to the shape of the curve, further suggesting that the mesoporous hysteresis loop was type H_3_. The specific surface area was 23.9 m^2^·g^−1^ for the nanoparticles, 20.8 m^2^·g^−1^ for the urchin-like microspheres, and 9.1 m^2^·g^−1^ for the nanoplatelets. 

**Figure 5:**
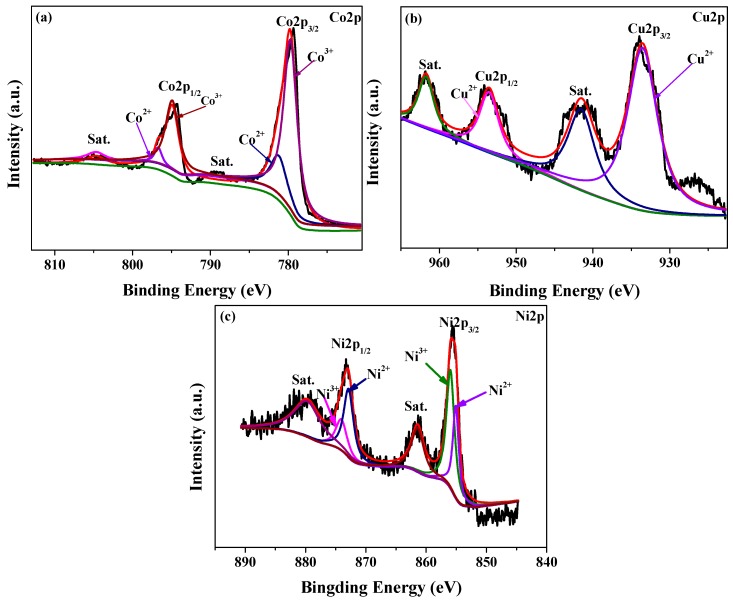
XPS spectra of the Ni_0.5_Cu_0.5_Co_2_O_4_ nanoplatelets: Co2p (**a**), Cu2p (**b**) and Ni 2p (**c**).

To gain a deeper insight into the electronic structure of the surface and the valence state of the corresponding elements, XPS analysis was performed on the Ni_0.5_Cu_0.5_Co_2_O_4_ nanoplatelets catalyst, and the results are shown in Figure 5. Two deconvoluted peaks at 796.7 and 794.9 eV were observed in the Co 2p_1/2_ region, while those at 781.5 and 779.6 eV were observed in the Co 2p_3/2_ region. The first and third peaks can be assigned to the Co^2+^ state, while the second and fourth peaks are ascribed to the Co^3+^ state. Notably, the spin-orbit splitting is 15.2 and 15.3 eV for the Co^2+^ and Co^3+^ doublets, respectively, implying that these Co species are not cobalt hydroxides but cobalt oxides [33]. The shake-up satellite peaks at 804.6 and 788.9 eV were very weak, indicating that there is a smaller portion of Co^2+^ in the sample [34]. There were four peaks in the spectrum of Cu. The peaks at 961.7 and 941.5 eV are assigned to satellite peaks, and those at 953.8 and 933.7 eV are indexed to the Cu 2p_1/2_ and Cu 2p_3/2_ peak, respectively, demonstrating that the Cu element was present as Cu^2+^. In the spectrum of Ni 2p_1/2_, two deconvoluted peaks at 873.9 and 872.8 eV were indexed to the Ni^3+^ and Ni^2+^ state, respectively. Similarly, two deconvoluted peaks at 855.9 and 855.2 eV in the Ni 2p_3/2_ region can be indexed to the Ni^3+^ and Ni^2+^ state, respectively. These observations are in line with the XPS data of CuCo_2_O_4_ [35] and NiCo_2_O_4_ [36]. We have also added the XPS spectra of the physical mixture of CuCo_2_O_4_ and NiCo_2_O_4_ in Figure S4. By analyzing the surface composition and chemical state, it is found that the relative contents of Ni^2+^ and Co^2+^ of the mixture are different from those of Ni_0.5_Cu_0.5_Co_2_O_4_ nanoplatelets (see Table S1). 

### 3.2. Catalytic Tests

**Figure 6:**
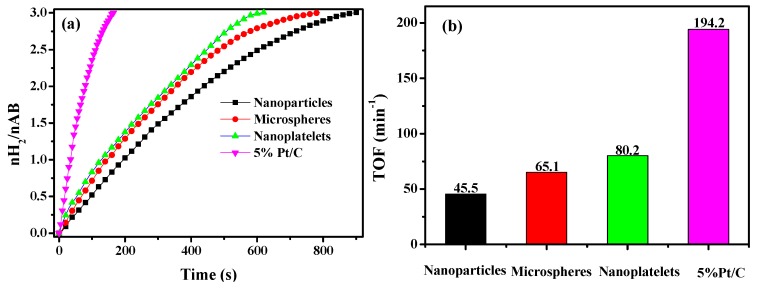
Hydrogen evolution from AB solution when different Ni_0.5_Cu_0.5_Co_2_O_4_ nanocomposites and commercial 5% Pt/C were used (**a**) and corresponding TOF (**b**).

To compare the catalytic performance of the Ni_0.5_Cu_0.5_Co_2_O_4_ catalysts with different morphology, hydrolysis reactions of AB catalyzed by different Ni_0.5_Cu_0.5_Co_2_O_4_ nanocomposites, as well as the commercial Pt/C catalyst, were performed and the results are presented in Figure 6. Clearly, all of these catalysts were active in AB hydrolysis, and the molar ratio of generated hydrogen to AB at the end of the hydrolytic reaction was 3, which means that the hydrolytic efficiency was 100%. As shown in Figure 6b, the TOF value was 44.5 mol_hydrogen_·min^−1^·mol^−1^_cat_ for Ni_0.5_Cu_0.5_Co_2_O_4_ nanoparticles and 65.1 mol_hydrogen_·min^−1^·mol^−1^_cat_ for Ni_0.5_Cu_0.5_Co_2_O_4_ microspheres consisting of nanowires. In contrast, the TOF value for the Ni_0.5_Cu_0.5_Co_2_O_4_ nanoplatelets reached 80.2 mol_hydrogen_·min^−1^·mol^−1^_cat_. Evidently, the catalytic activity of the Ni_0.5_Cu_0.5_Co_2_O_4_ nanoplatelets is much lower than that of the Pt/C catalyst. However, they are still attractive owing to the low cost and relatively high catalytic activity. Notably, at the late stages of the hydrolysis reaction, there is a deviation from the linear dependence of the hydrogen volume on the reaction time, which may be caused by the external diffusion limitation at a low concentration of AB [37]. The other possible reason for this is the deactivation of the catalyst [38]. We have calculated the BET surface area normalized TOF, which is 380.8 mol_hydrogen_·min^−1^·mol^−1^_cat_·m^−2^ for the Ni_0.5_Cu_0.5_Co_2_O_4_ nanoparticles, 626.0 mol_hydrogen_·min^−1^·mol^−1^_cat_·m^−2^ for the urchin-like Ni_0.5_Cu_0.5_Co_2_O_4_ microspheres and 1762.6 mol_hydrogen_·min^−1^·mol^−1^_cat_·m^−2^ for the Ni_0.5_Cu_0.5_Co_2_O_4_ nanoplatelets, respectively.

**Figure 7:**
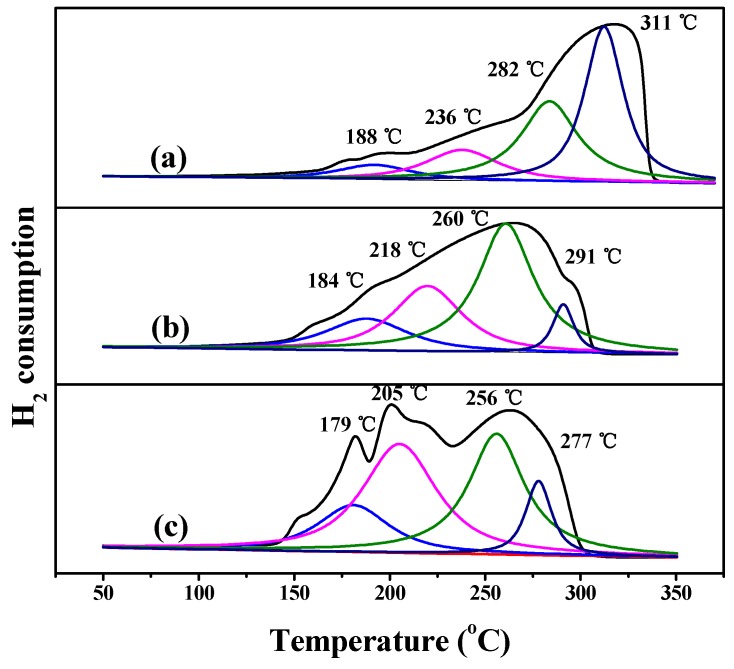
Deconvoluted H_2_–TPR profiles of different Ni_0.5_Cu_0.5_Co_2_O_4_ nanocomposites: nanoparticles (**a**), microspheres (**b**), and nanoplatelets (**c**).

Notably, among these three Ni_0.5_Cu_0.5_Co_2_O_4_ nanostructures, the Ni_0.5_Cu_0.5_Co_2_O_4_ nanoplatelets exhibited the highest catalytic activity despite having the lowest BET surface area. According to the literature, metallic Ni [16], Co [17], and Cu [18] are all active to AB hydrolysis. However, the catalytic activity of single metal of Ni, Co, or Cu is not so high. Their performance can be improved by alloying two or three of them. In our previous studies on AB hydrolysis catalyzed by oxide-based catalysts [35,36], we found that the corresponding alloy on the surface of the catalyst, which is generated by the reduction of oxides with AB, will act as an active species. Thus, the formation rate of active species on the oxide surface will significantly affect the catalytic behavior. In other words, the catalytic activity of these oxide-based catalysts is highly dependent on their reducibility. The redox properties of differently shaped Ni_0.5_Cu_0.5_Co_2_O_4_ nanocomposites were investigated by H_2_–TPR and the results are shown in Figure 7. There are four deconvoluted peaks in the H_2_-TPR profiles of Ni_0.5_Cu_0.5_Co_2_O_4_ nanoplatelets. The first two peaks are centered at 179 and 205 °C, and are associated with the transformation of Cu^2+^ to Cu^+^ and Cu^+^ to metallic Cu, respectively [39]. According to the literature, the reduction of Co^3+^ occurs at around 250 °C. Thus, the peak at 256 °C is believed to be related to the reduction of Co_2_O_4_^2−^ [40]. A relatively weak peak is observed at around 277 °C, which overlapped with the wide peak at 264 °C. This may be related to the reduction of Ni^2+^ to metallic Ni [40]. In contrast, all the corresponding deconvoluted peaks in the H_2_–TPR profiles of the Ni_0.5_Cu_0.5_Co_2_O_4_ nanoparticles and microspheres are shifted positively, demonstrating that the respective reduction process becomes difficult. In particular, the reduction peak of Ni(II) of Ni_0.5_Cu_0.5_Co_2_O_4_ nanoparticles is shifted to higher values (ca. 34 °C) compared with nanoplatelets, indicating that it is relatively difficult to reduce Ni(II) to Ni(0). According to the literature [41], Ni could markedly enhance the catalytic performance of Co and Cu in AB hydrolysis when Ni is combined with Co and Cu. In our case, it is likely that the poor reducibility of the Ni(II) in the Ni_0.5_Cu_0.5_Co_2_O_4_ nanoparticles results in their lower catalytic performance. For comparison, the H_2_–TPR curve of the physical mixture of CuCo_2_O_4_ and NiCo_2_O_4_ is shown in Figure S5. Evidently, the corresponding reduction process of the mixture takes place at a higher temperature than that of the Ni_0.5_Cu_0.5_Co_2_O_4_ nanoplatelets, verifying that the mixture is more difficult to reduce than the Ni_0.5_Cu_0.5_Co_2_O_4_ nanoplatelets.

To compare the catalytic behavior of our Ni_0.5_Cu_0.5_Co_2_O_4_ nanocomposites with other noble-metal-free catalysts, we show their TOF values in Table 1. The TOF of our Ni_0.5_Cu_0.5_Co_2_O_4_ nanoplatelet catalysts is one of the highest TOF values ever reported for those noble-metal-free catalysts. It should be mentioned that Cu_0.6_Ni_0.4_Co_2_O_4_ nanowires exhibit better catalytic performance than our Ni_0.5_Cu_0.5_Co_2_O_4_ nanoplatelets in the present study. There are two possible reasons for that. Firstly, the relative contents of Ni and Cu play a crucial role in determining the catalytic activity. In this work, the molar ratio of Cu to Ni in Ni_0.5_Cu_0.5_Co_2_O_4_ nanoplatelets is lower than that in Cu_0.6_Ni_0.4_Co_2_O_4_ nanowires. In addition, the morphology of the nanocatalysts will significantly influence their activity. According to the literature, the speed of electron transfer in one-dimensional nanowires is much faster, which may result in the fast hydrolysis of AB. 

**Figure 8:**
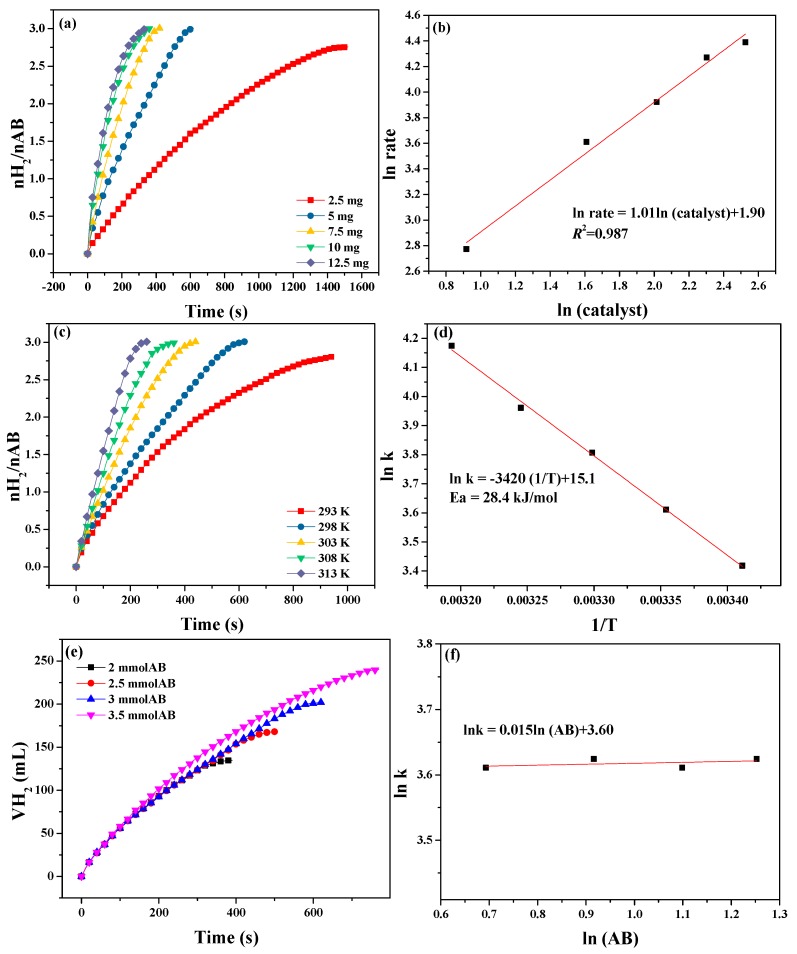
(**a**) Hydrogen evolution at different catalyst dosages (T = 298 K, AB dosage = 3.0 mmol); (**b**) the dependence of the logarithmic values of the hydrogen releasing rate on the logarithmic values of catalyst mass (T = 298 K, AB dosage = 3.0 mmol); (**c**) hydrogen evolution at reaction temperatures of 293–313 K (AB dosage = 3.0 mmol, catalyst weight = 5.0 mg); (**d**) ln k versus 1/T; (**e**) hydrogen evolution at different AB dosages (T = 298 K, catalyst weight = 5.0 mg); and (**f**) the relationship between the logarithmic values of the hydrogen generation rate constant and the logarithmic values of AB dosage.

To study the dependence of the hydrogen production by AB hydrolysis on the dosage of the catalyst, different amounts of Ni_0.5_Cu_0.5_Co_2_O_4_ nanoplatelets were used in the AB hydrolysis reaction, and the results are shown in Figure 8a. The rate of hydrogen release increased when more catalyst was used. To obtain more detailed information, the relationship between the logarithmic values of the catalyst dosage and the corresponding logarithmic values of the hydrogen production rate are shown in Figure 8b. The slope of the fitting line is 1.01, indicating that AB hydrolysis is a first-order reaction related to the catalyst mass. This observation is consistent with the results of Lu et al. [33]. Accordingly, it is easy for us to adjust the rate of hydrogen generation by tuning the catalyst dosage. The dependence of the hydrogen production rate on the reaction temperature was also investigated. The data presented in Figure 8c reveal that the hydrogen evolution rate increases at a higher temperature. The relationship between the logarithm of the rate constants and the reciprocal of the reaction temperatures is shown in Figure 8d. According to the Arrhenius equation, the apparent activation energy was 28.4 kJ·mol^−1^. We also calculated the apparent activation energy for the Ni_0.5_Cu_0.5_Co_2_O_4_ nanoparticles and urchin-like Ni_0.5_Cu_0.5_Co_2_O_4_ microspheres, which are 43.2 and 29.5 kJ·mol^−1^, respectively (see Figure S6). The evaluation of the effect of the AB dosage on the catalytic hydrogen production was performed at 298 K. The data shown in Figure 8e reveal that the initial hydrogen production rates remained almost unchanged with the increase in the AB dosage. The relationship between the logarithmic values of the hydrogen generation rate constant and that of the AB weight is depicted in Figure 8f. The slope of the fitting line is 0.015, very close to 0, which suggests that AB hydrolysis is a zero-order reaction for AB. This finding is consistent with the reported results [33].

Based on the above analysis, the kinetic equation for AB hydrolysis catalyzed by Ni_0.5_Cu_0.5_Co_2_O_4_ nanoplatelets can be deduced from the concentration of the catalyst and AB as follows:

(2) $$r=-\frac{d\left[AB\right]}{dt}=k{\left[catalyst\right]}^{1.01}{\left[AB\right]}^{0.015}\approx k^{\prime }{\left[catalyst\right]}^{1.01},$$

(3) $$k^{\prime }=A\exp\left(-\frac{Ea}{RT}\right)\to \ln k^{\prime }=\mathrm{lnA}-\frac{Ea}{RT},$$

where, *r* is reaction rate (mol·L^−1^·s^−1^), *k* is reaction rate constants (s^−1^), *A* is pre-exponential factor (s^−1^), *Ea* is activation energy (J·mol^−1^), *R* is ideal gas constant (J·k^−1^·mol^−1^).

In Equation (3), ln *A* equals the intercept of the fitting line in Figure 8d. Thus, the rate law can be expressed as in the following equation:

(4) $$r=-\frac{d\left[AB\right]}{dt}=3612823\exp\left(-\frac{3416}{T}\right){\left[catalyst\right]}^{1.01}.$$

The stability and reusability of the catalyst are quite important in practical applications. Figure S7 depicts the hydrogen evolution at different catalytic cycles when the Ni_0.5_Cu_0.5_Co_2_O_4_ nanoplatelets act as catalysts. After five catalytic cycles, there is only a slight activity loss, hinting that the Ni_0.5_Cu_0.5_Co_2_O_4_ nanoplatelets possess relatively high stability and good reusability. The Ni_0.5_Cu_0.5_Co_2_O_4_ nanoplatelets after the catalytic reaction were checked with SEM, XRD, and XPS, and the results are shown in Figures S8 and S9. The SEM image in Figure S8a indicates that the architecture of the nanoplatelets of the sample was maintained, but some of these nanoplatelets aggregate together. In addition, there was no significant difference between the size of the nanoplatelets before and after the catalytic reaction. The XRD pattern in Figure S8b demonstrates that, besides the characteristic peaks of the Ni_0.5_Cu_0.5_Co_2_O_4_ nanocomposite, characteristic peaks of the CoCu and CuNi alloys are also observed. The XPS results in Figure S9 further confirm that Co(0), Cu(0), and Ni(0) are formed on the surface of the used catalysts. Considering these results together, it is rational to conclude that the active alloys are formed on the surface of the sample, which will catalyze AB to release hydrogen in the catalytic process.

## 4. Conclusions

In summary, we prepared three Ni_0.5_Cu_0.5_Co_2_O_4_ nanocomposites with different morphology, namely nanoplatelets, nanoparticles, and urchin-like microspheres composed of nanowires. In AB hydrolysis, the Ni_0.5_Cu_0.5_Co_2_O_4_ nanoplatelets exhibited the best catalytic performance, with a TOF of 80.2 mol_hydrogen_·min^−1^·mol^−1^_cat_ despite their low specific surface area. It was also found that, in contrast to the Ni_0.5_Cu_0.5_Co_2_O_4_ nanoparticles and microspheres, the Ni_0.5_Cu_0.5_Co_2_O_4_ nanoplatelets are more readily reduced, leading to the fast formation of active species in AB hydrolysis, which results in the high catalytic performance. These findings provide deeper insight into the design of high-performance oxide-based catalysts for AB hydrolysis. Considering their low cost and high catalytic activity, the Ni_0.5_Cu_0.5_Co_2_O_4_ nanoplatelets are a strong candidate catalyst for the production of hydrogen through AB hydrolysis in practical applications.

## Figures and Tables

**Table 1:** TOF and Ea of some representative non-noble catalysts and commercial Pt/C toward AB hydrolysis.

Catalysts	TOF (mol_hydrogen_·min^−1^·mol^−1^_cat_)	Ea (kJ·mol^−1^)	Reference
5% Pt/C	194.2	19.1	This Work
Cu_0.6_Ni_0.4_Co_2_O_4_ nanowires	119.5	33.91	[33]
NiCoP/OPC-300	95.2	38.9	[42]
Ni-ZIF8	85.7	28.0	[43]
Co_x_Cu_1__−__x_Co_2_O_4_@ Co_y_Cu_1__−__y_Co_2_O_4_ yolk–shell microspheres	81.8	24.97	[44]
Ni_0.5_Cu_0.5_Co_2_O_4_ nanoplatelets	80.2	28.4	This work
CuCo_2_O_4_	73.4	/ ^a^	[45]
Cu_0.8_Co_0.2_O-GO	70.0	45.53	[23]
Ni_0.5_Cu_0.5_Co_2_O_4_ microspheres	65.1	29.5	This work
Co_0.8_Cu_0.2_MoO_4_ microspheres	55.0	39.6	[38]
Ni_0.5_Cu_0.5_Co_2_O_4_ nanoparticles	45.5	43.2	This work
Ni_2_P NPs	40.4	44.6	[46]
Co/PEI-GO	39.9	28.2	[47]
MoO_3_-doped MnCo_2_O_4_	26.4	34.24	[48]
Cu@FeCoNi/graphene	20.93	31.82	[49]
CuCo@MIL-101	19.6	/	[50]
GeCH_3_	18.1	/	[51]
Cu_0.33_Fe_0.67_	13.9	43.2	[52]
Ni/SiO_2_	13.2	34 ± 2	[53]
Cu_0.3_@Fe_0.1_Co_0.6_ core-shell nanoparticles	10.5	38.75	[54]
PSMA-Ni	10.1	32 ± 2	[55]
CuCo/rGO	9.1	/	[56]
Ni_2_P	8.1	/	[57]

^a^: No data are reported.

## Supplementary Materials

The following are available online at https://www.mdpi.com/2079-4991/9/9/1334/s1, Figure S1: SEM images of the physical mixture of CuCo_2_O_4_ and NiCo_2_O_4_ (a, b) and EDS patterns on some selected nanoplatelets; Figure S2: TEM image of nanoparticles (a), HRTEM image of nanoparticles (b), TEM image of urchin-like microspheres (c), HRTEM image of urchin-like microspheres (d); Figure S3: N_2_ absorption-desorption isotherms curves of nanoparticles (a), urchin-like microspheres (b) and nanoplatelets (c); Figure S4: XPS spectra of the CuCo_2_O_4_ and NiCo_2_O_4_ mixture; Figure S5: H_2_–TPR curve of the mixture of CuCo_2_O_4_ and NiCo_2_O_4_; Figure S6: Hydrogen evolution at different temperature (a,c) and the calculation of the activation energy for different catalysts (b,d); Figure S7: Hydrogen evolution at different recycle number when the CuCo_2_O_4_/NiCo_2_O_4_ nanoplatelets act as catalysts; Figure S8: SEM image (a) and the XRD pattern (b) of the used Ni_0.5_Cu_0.5_Co_2_O_4_ nanoplatelets after catalytic reaction; Figure S9: XPS spectra of CuCo_2_O_4_/NiCo_2_O_4_ nanoplatelets after catalytic reaction; Table S1: Comparison of the relative contents of Ni^2+^ and Co^2+^ on the surface of the composition and mixture.
