# Supplementary material for: Ni0.5Cu0.5Co2O4 Nanocomposites, Morphology, Controlled Synthesis, and Catalytic Performance in the Hydrolysis of Ammonia Borane for Hydrogen Production
Source: Nanomaterials (Basel). 2019 Sep 18;9(9):1334. doi: 10.3390/nano9091334 (PMC6781025; doi:10.3390/nano9091334)
Supplement: Supplementary file 1 [file nanomaterials-09-01334-s001.pdf]

nanomaterials

Article

$Ni_{0.5}Cu_{0.5}Co_2O_4$ Nanocomposites, Morphology, Controlled Synthesis, and Catalytic Performance in the Hydrolysis of Ammonia Borane for Hydrogen Production

Yufa Feng [1], Jin Zhang [1], Huilong Ye [1], Liling Li [2], Huize Wang [1], Xian Li [1], Xibin Zhang [1] and Hao Li [1,*]

[1] School of Chemistry and Materials Engineering, Huizhou University, Huizhou 516007, China; yufafeng@126.com (Y.F.); eeedwardjin@163.com (J.Z.); yehuilong6364@163.com (H.Y.); whz@hzu.edu.cn (H.W.); lixian2020@126.com (X.L.); zxbin1@163.com (X.Z.)

[2] Department of Pharmacy, Huizhou Health Sciences Polytechnic, Huizhou 516025, China; 13692842548@163.com (L.L.)

* Correspondence: lihao180@126.com (H.L.); Tel.: +86-752-2527229

Received: 16 August 2019; Accepted: 10 September 2019; Published: date

Supplementary Materials:

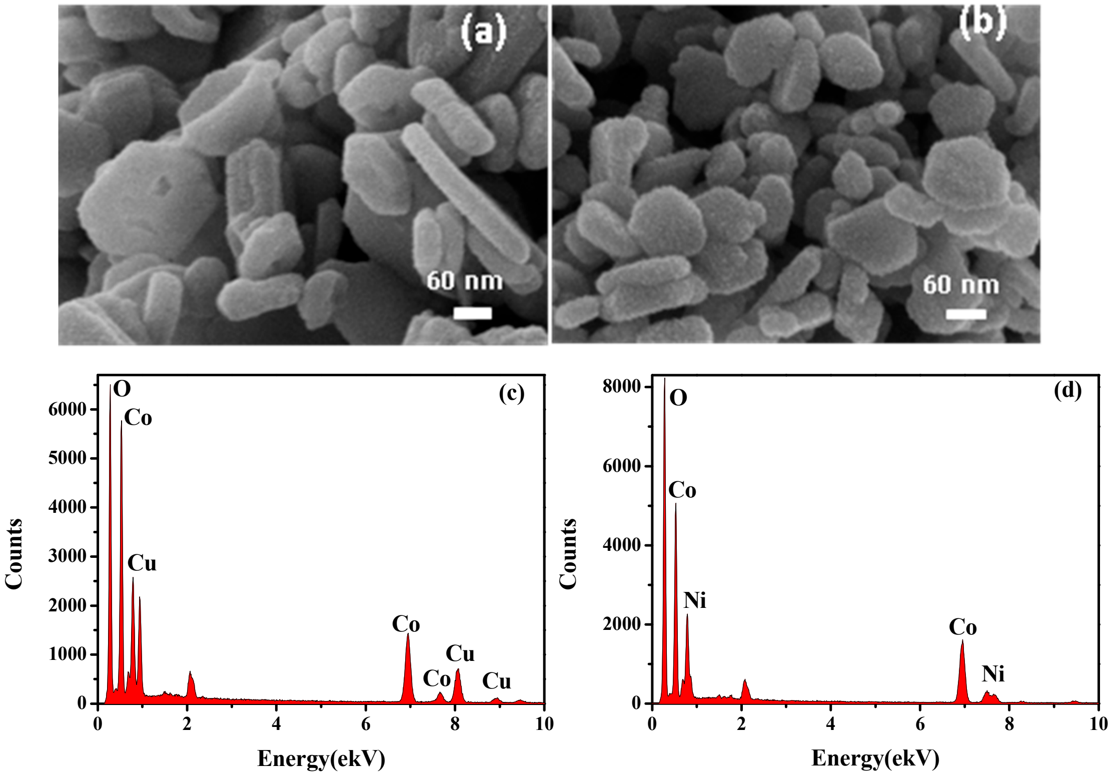

Figure S1 SEM images of the physical mixture of $CuCo_2O_4$ and $NiCo_2O_4$ (**a**,**b**) and EDS patterns on some selected nanoplatelets.

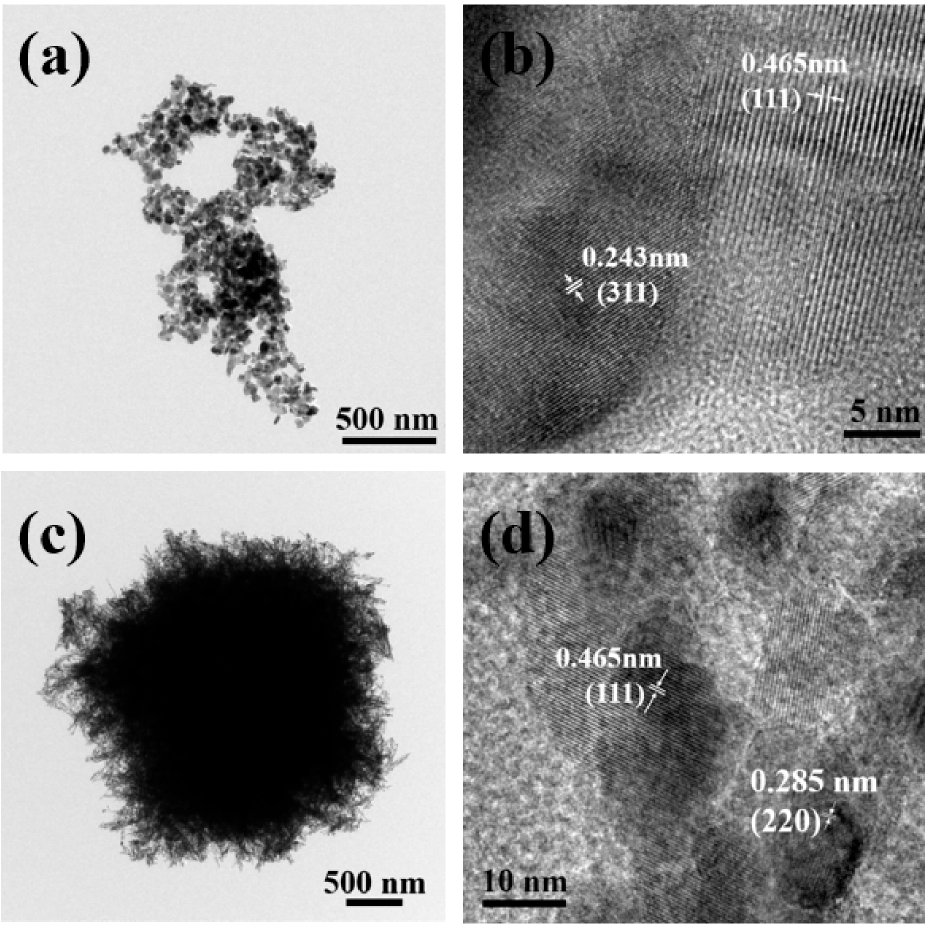

Figure S2. TEM image of nanoparticles (**a**), HRTEM image of nanoparticles (**b**), TEM image of urchin-like microspheres (**c**), HRTEM image of urchin-like microspheres (**d**).

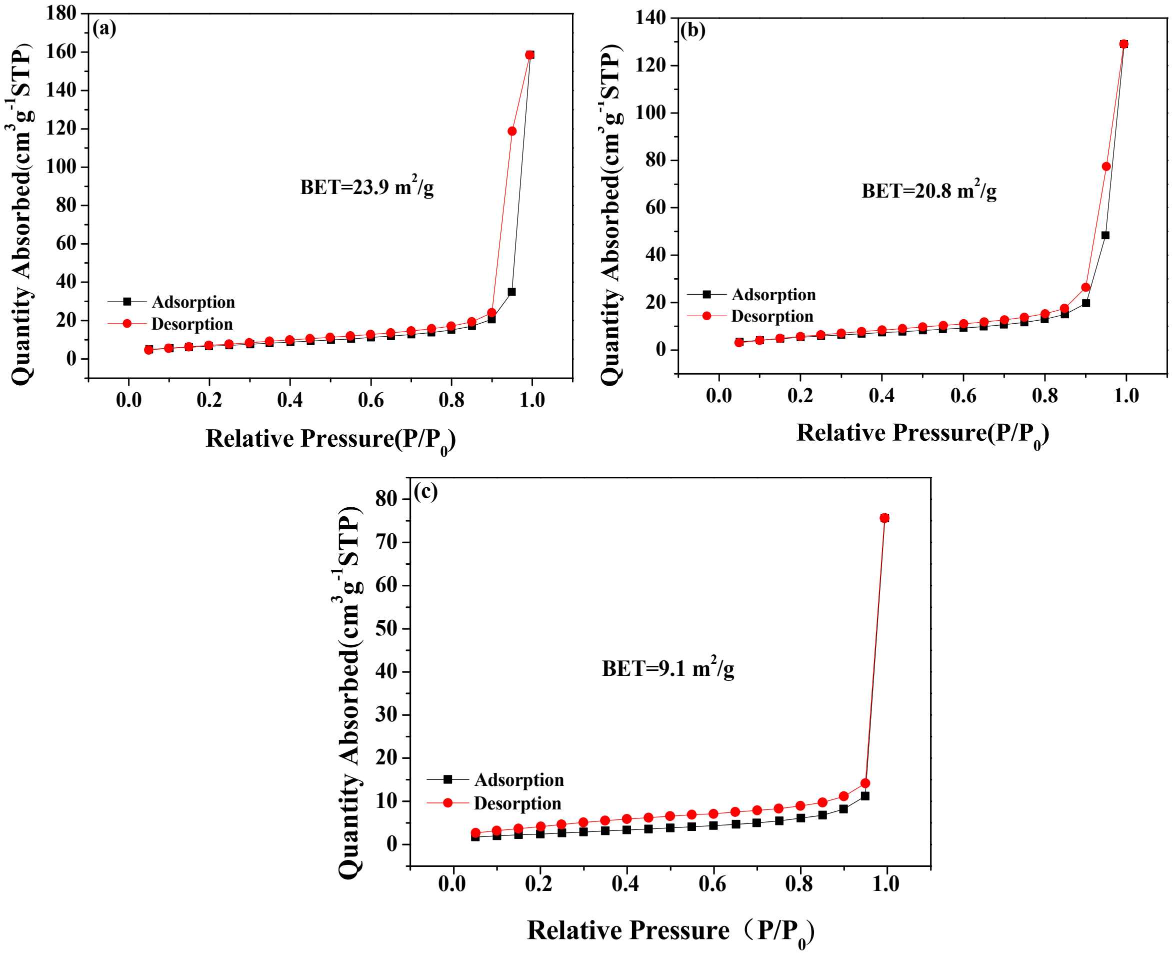

Figure S3. N_2 absorption-desorption isotherms curves of nanoparticles (a), urchin-like microspheres (b) and nanoplatelets (c).

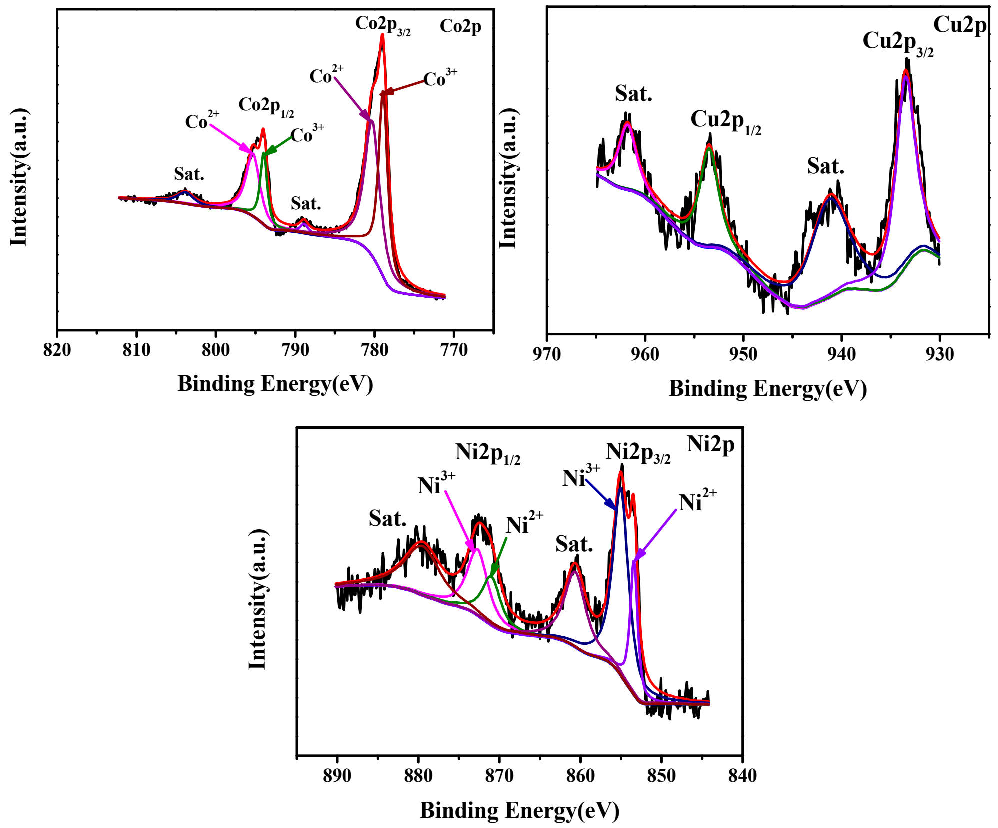

Figure S4 XPS spectra of the $CuCo_2O_4$ and $NiCo_2O_4$ mixture

Table S1 Comparison of the relative contents of Ni^{2+} and Co^{2+} on the surface of the composition and mixture

Catalyst	Ni^{2+}/Ni(%)	Ni^{3+}/Ni(%)	Co^{2+}/Co(%)	Co^{3+}/Co(%)
Composition	35.5	64.5	27.7	72.3
Mixture	23.8	76.2	42.1	57.9

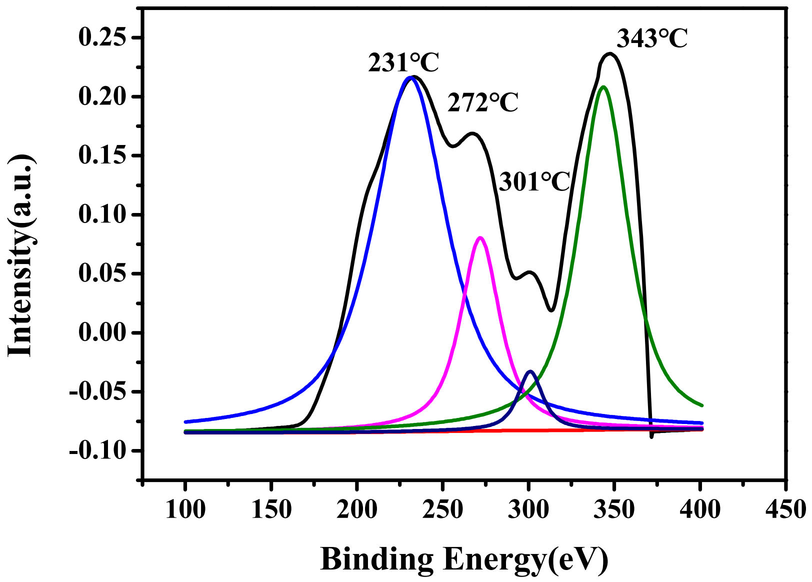

Figure S5 H_2-TPR curve of the mixture of $CuCo_2O_4$ and $NiCo_2O_4$.

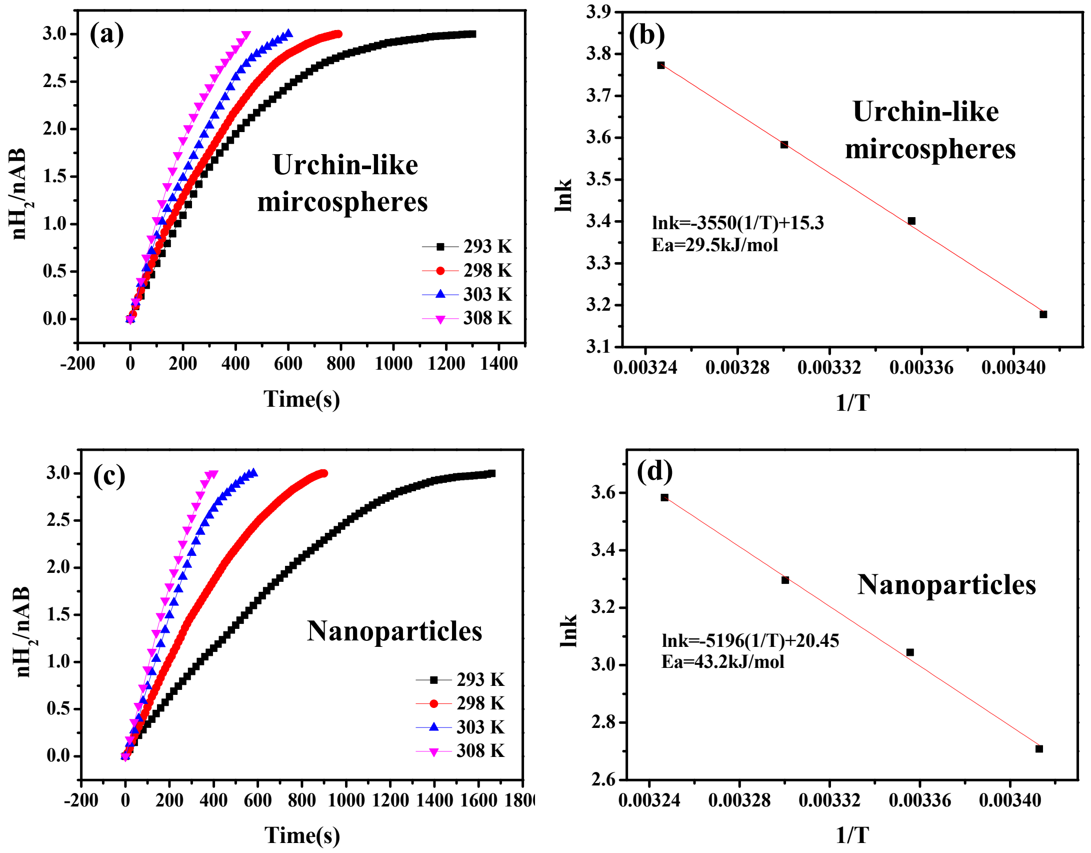

Figure S6. Hydrogen evolution at different temperature (**a**,**c**) and the calculation of the activation energy for different catalysts (**b**,**d**).

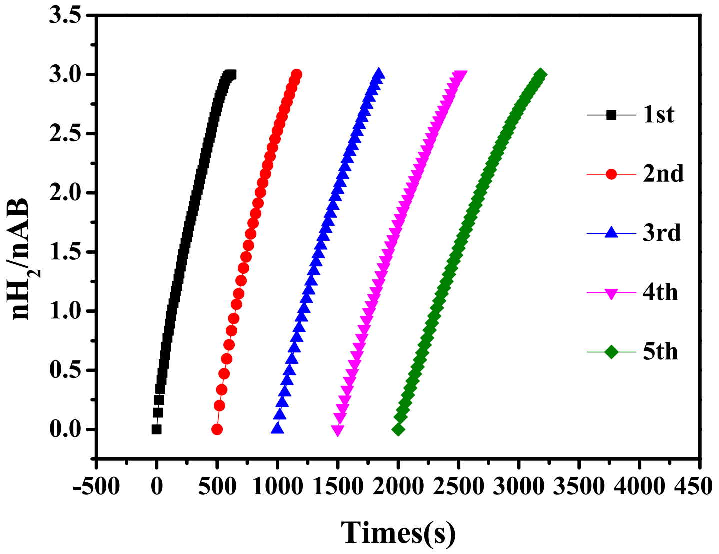

Figure S7. Hydrogen evolution at different recycle number when the $CuCo_2O_4/NiCo_2O_4$ nanoplatelets act as catalysts.

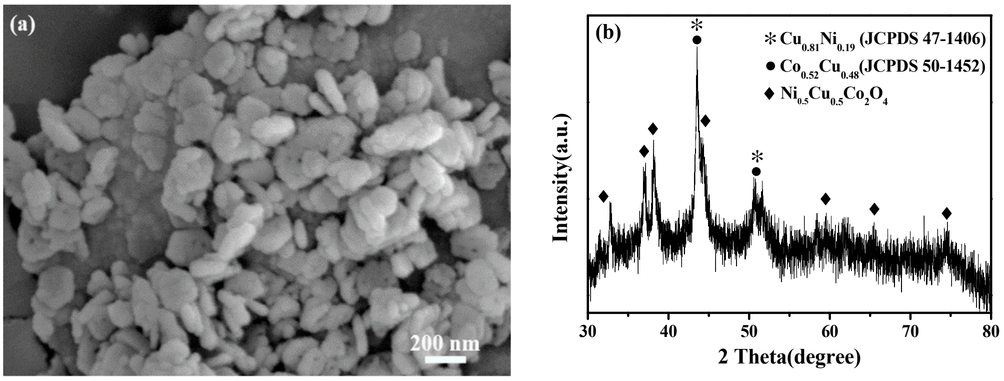

Figure S8. SEM image (**a**) and the XRD pattern (**b**) of the used $Ni_{0.5}Cu_{0.5}Co_2O_4$ nanoplatelets after catalytic reaction.

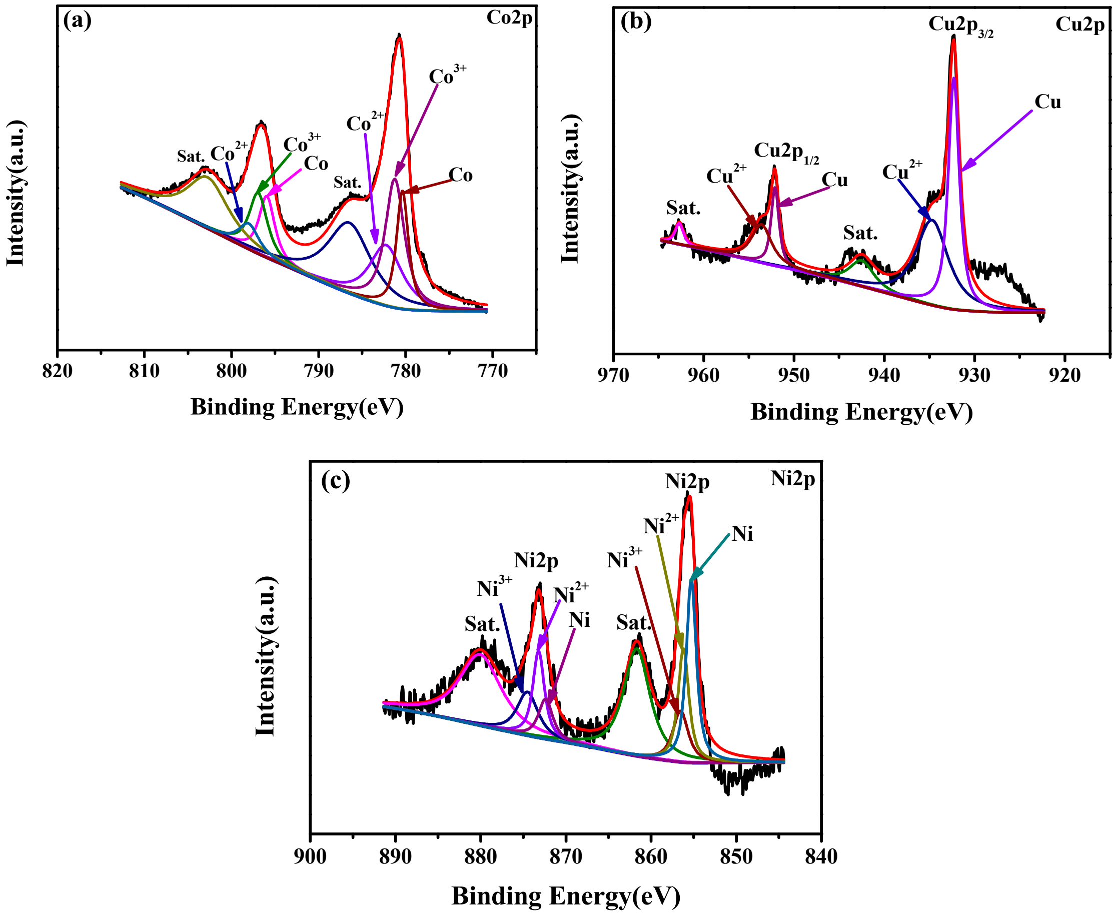

Figure S9 XPS spectra of $CuCo_2O_4/NiCo_2O_4$ nanoplatelets after catalytic reaction.

© 2019 by the authors. Submitted for possible open access publication under the terms and conditions of the Creative Commons Attribution (CC BY) license (http://creativecommons.org/licenses/by/4.0/).
